# Large HERCs Function as Tumor Suppressors

**DOI:** 10.3389/fonc.2019.00524

**Published:** 2019-06-18

**Authors:** Taiane Schneider, Arturo Martinez-Martinez, Monica Cubillos-Rojas, Ramon Bartrons, Francesc Ventura, Jose Luis Rosa

**Affiliations:** Departament de Ciències Fisiològiques, IDIBELL, Universitat de Barcelona, Barcelona, Spain

**Keywords:** ubiquitin, ERK, RAF, proliferation, p53, oligomerization, NEURL4

Ubiquitin ligases regulate numerous cellular processes, including tissue homeostasis, cellular metabolism, and cell cycle progression. These enzymes recognize, interact with and ubiquitylate specific substrates. Homologous to the E6-AP carboxyl terminus (HECT) and regulator of chromosome condensation 1 (RCC1)-like domain-containing proteins (HERCs) belong to the family of HECT ubiquitin ligases. There are six human HERCs which can be divided into two subgroups: large HERCs (HERC1-2) and small HERCs (HERC3-6). Alterations in the function of large HERCs are associated with serious pathologies such as neurological disorders. Mutations in human HERC1 have been associated with overgrowth, intellectual disability and some autistic features; while mutations in HERC2 have been identified as the cause of a neurodevelopmental disorder with similarities to Angelman syndrome and autism (1).

Large HERCs are also known to be involved in human cancers. Mutations in HERC1 and HERC2 have been detected in leukemia cells (T-cell acute lymphoblastic leukemia (T-ALL) for HERC1 and T-cell prolymphocytic leukemia (T-PLL) for HERC1 and HERC2) and in breast cancer tumors. Frameshift mutations in HERC2 have been reported in gastric and colorectal carcinomas with microsatellite instability. HERC1 has been associated with non-melanoma skin cancer through regulation of E6-mediated BAK degradation, and the HERC2 locus with cutaneous melanoma and uveal melanoma (2–6).

More recent studies conducted by the Human Protein Atlas (7) confirm the involvement of large HERCs in cancer (Figure 1A). Patients with kidney cancer, head and neck cancer, and pancreatic cancer show greater survival when their HERC1 expression levels are higher (Figure 1A, left). Similarly, greater survival is observed in renal cancer patients when HERC2 expression are higher (Figure 1A, right). These expression data together with the observations described above associating inactivating mutations of large HERCs with different types of cancer, suggest that these genes exhibit a tumor suppressor function.

**Figure 1:**
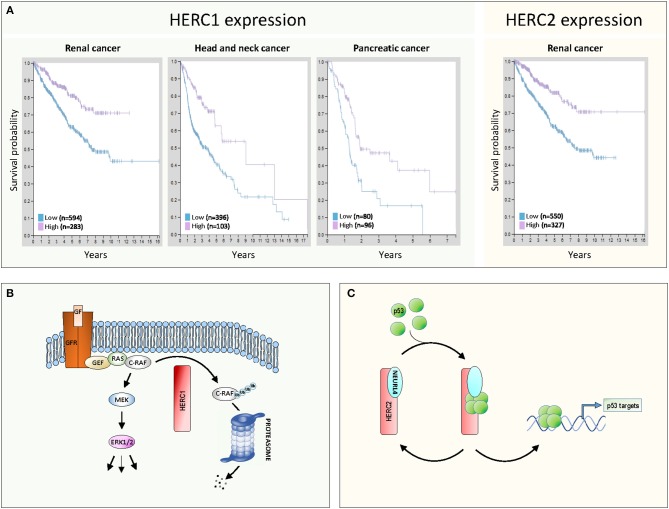
Large HERCs involved in cancer. **(A)** Gene expression of HERC1 and HERC2 in human cancers. Data were obtained from the Human Protein Atlas (7). The Kaplan-Meier plots show the results from analysis of the correlation between patient survival and mRNA expression level for HERC1 (www.proteinatlas.org/ENSG00000103657-HERC1/pathology) and HERC2 (www.proteinatlas.org/ENSG00000128731-HERC2/pathology). Patients were classified into one of two groups based on the level of expression: low or high. **(B,C)** Molecular mechanisms of action of large HERCs as tumor suppressors: **(B)** HERC1 controls the ERK signaling pathway, targeting C-RAF for proteasome-dependent degradation; **(C)** the HERC2-NEURL4 complex controls p53 transcriptional activity by regulating its oligomerization state. See details in the text.

Despite all this evidence, the cellular functions of large HERCs in cancer are poorly understood. Knowledge of these functions and the underlying molecular mechanisms should explain the role as tumor suppressors that genetic analysis suggests large HERCs play. Up to now, the role of large HERCs in cancer has mainly been attributed to the regulation of proteins involved in DNA damage response such as MSH2, XPA, RNF8, or BRCA1 (1). Recent studies further propose two novel molecular models for large HERCs that are compatible with their tumor suppressor function (Figures 1B,C).

The first model emerges from the study performed by Schneider et al. (8). Those authors demonstrate how HERC1 regulates ERK signaling. Classically, growth factors activate ERK through activation of their receptors and the GEF/RAS/RAF/MEK signaling pathway (Figure 1B). Gain of function of this pathway is associated with proliferation and tumorigenesis. Schneider et al. observe that HERC1 knockdown induces cellular proliferation associated with an increase in ERK activity. A specific rise in the amount of C-RAF accounts for this ERK activation. Pharmacological inhibitors and RNA interference assays confirmed that regulation of ERK activity by HERC1 is dependent on changes in C-RAF stability. HERC1 ubiquitylates C-RAF targeting it for proteasome-dependent degradation. When levels of HERC1 are low, C-RAF degradation decreases and its level is sufficient to activate the RAF/MEK/ERK signaling pathway and cell proliferation (Figure 1B). This model could account for the improved prognosis for cancer patients when levels of HERC1 expression are higher (Figure 1A).

The second model emerges from studies performed by Cubillos-Rojas et al. (9, 10). Those authors demonstrate how HERC2 regulates p53 activity. p53 is a transcription factor that regulates important cellular processes related to tumor suppression, including induction of senescence, apoptosis, and DNA damage response, as well as the inhibition of angiogenesis and cell migration. p53 tetramerization is a key step in its activation process and the regulation of this oligomerization is an important control point. Cubillos-Rojas et al. show that HERC2 interacts with the adaptor-like protein with six neuralized domains (NEURL4) and that this complex controls p53 transcriptional activity by regulation of its oligomerization state (Figure 1C). Furthermore, the studies demonstrate that regulation of p53 tetramerization by HERC2 is independent of proteasome activity. The role of the HERC2-NEURL4 complex in activating the tumor suppressor p53 could explain the improved prognosis for cancer patients when levels of HERC2 expression are higher (Figure 1A). According to this model, NEURL4 should also function as a tumor suppressor and in agreement with this, analysis of cancer genomics datasets from the cBioPortal shows a high frequency in NEURL4 deletions in prostate cancer (10).

These two models for large HERCs are compatible with a tumor suppressor function. This function is also compatible with their role in the DNA damage response, which could lead to a higher mutation burden (mutator phenotype). All these findings highlight the important physiological role of large HERCs and how their loss of function is associated with tumorigenesis. Knowing the mechanisms through which large HERCs regulate cellular processes may be helpful in identifying new prognostic markers and in designing more specific and efficient anti-cancer therapies.

## Author Contributions

TS, AM-M, MC-R, RB, FV, and JR interpreted data and wrote the manuscript. All authors read, edited, and approved the manuscript.

### Conflict of Interest Statement

The authors declare that the research was conducted in the absence of any commercial or financial relationships that could be construed as a potential conflict of interest.
